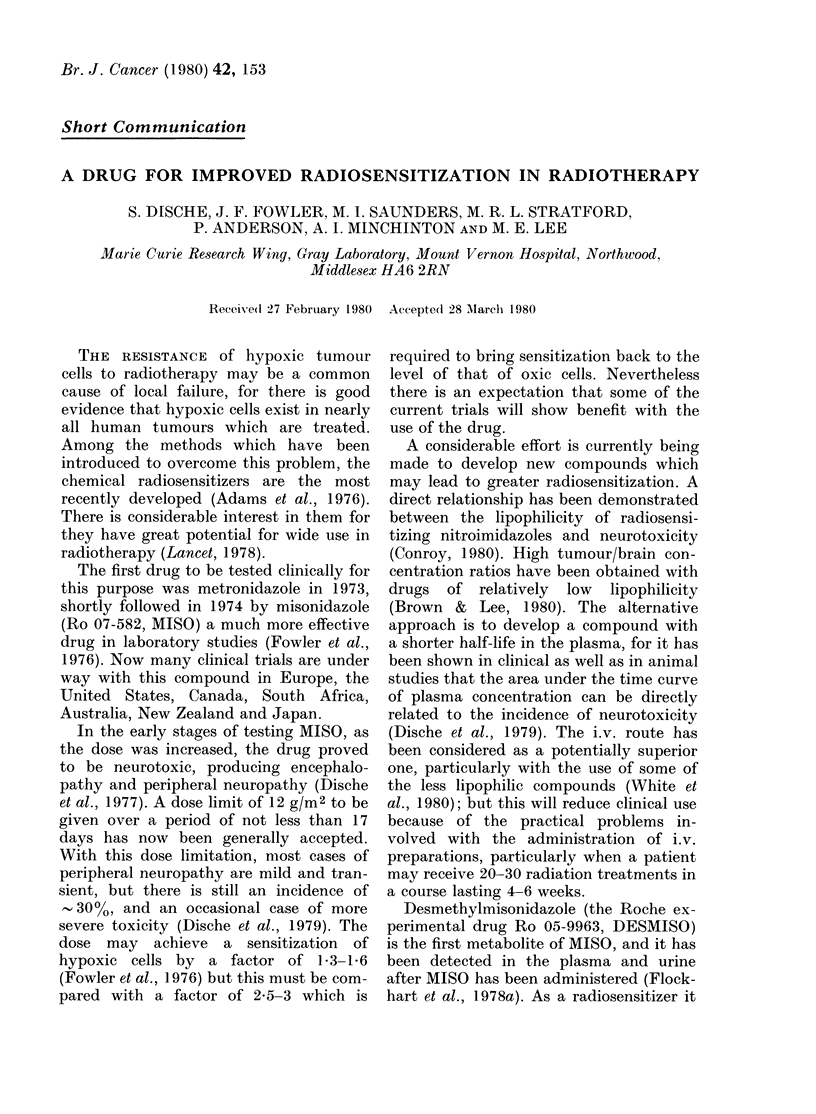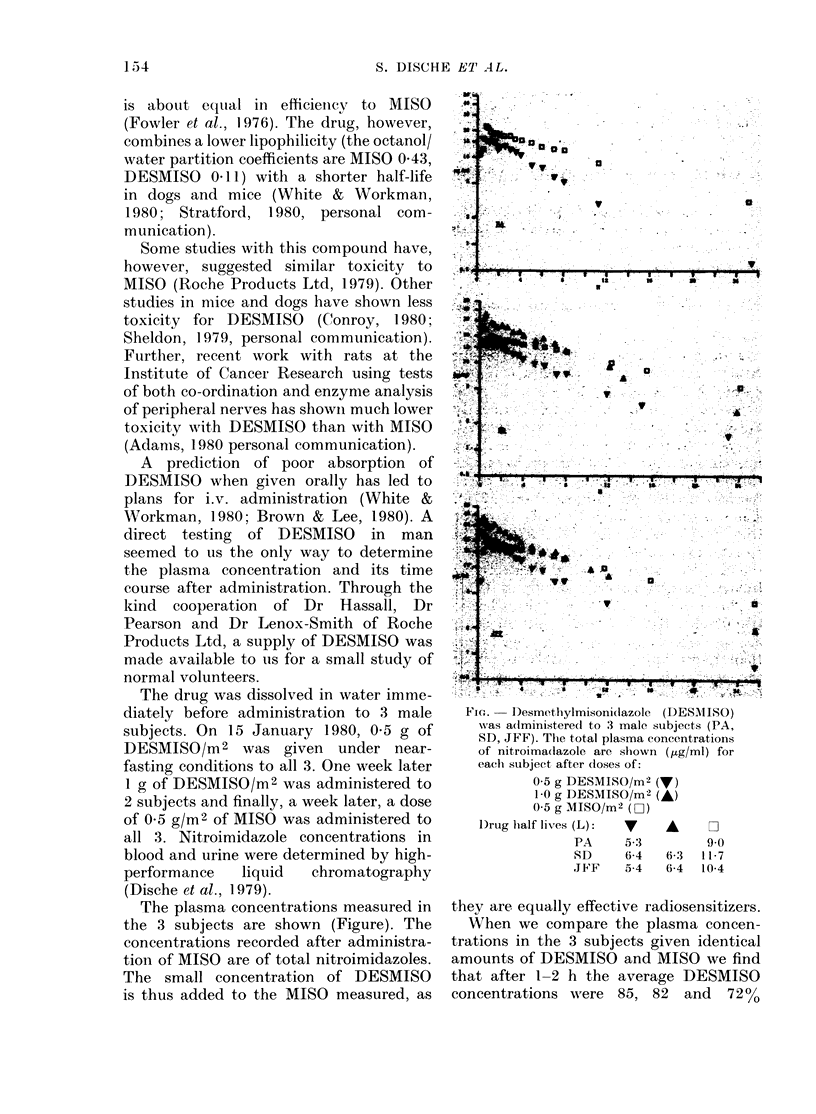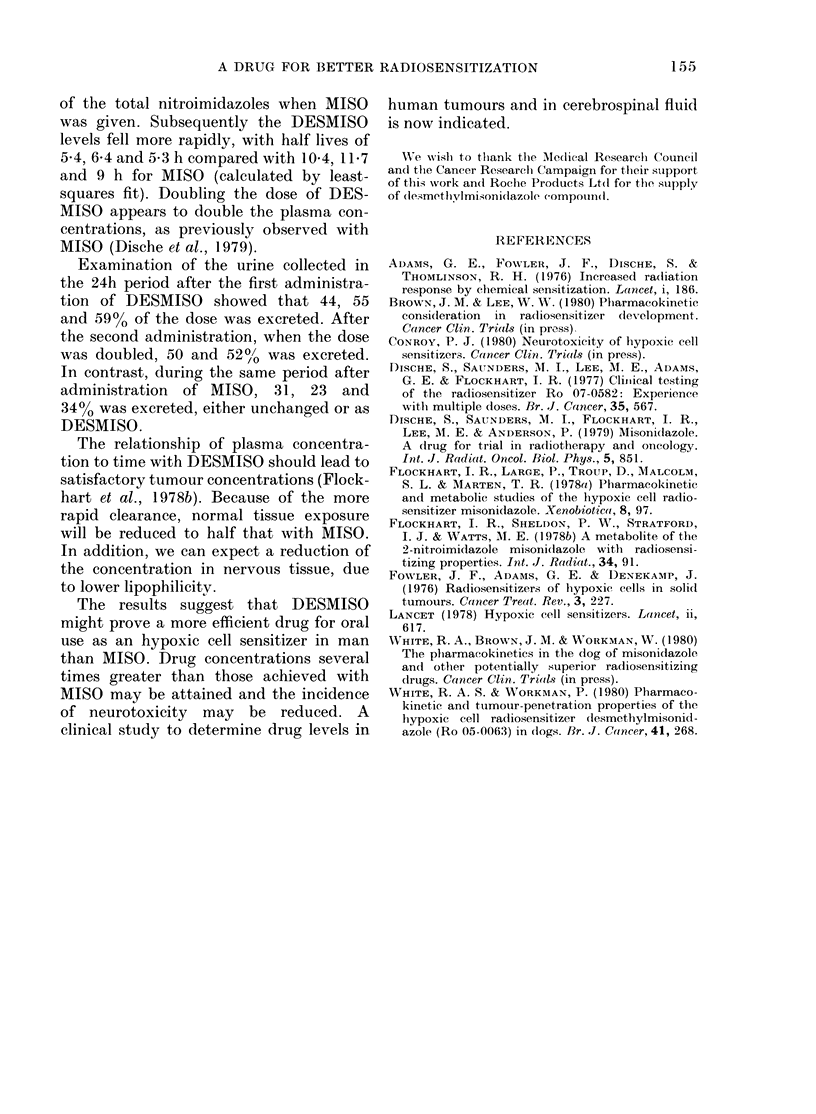# A drug for improved radiosensitization in radiotherapy.

**DOI:** 10.1038/bjc.1980.213

**Published:** 1980-07

**Authors:** S. Dische, J. F. Fowler, M. I. Saunders, M. R. Stratford, P. Anderson, A. I. Minchinton, M. E. Lee


*Br. J. Cancer* (1980) **42**, 153

***Short Communication***

### A DRUG FOR IMPROVED RADIOSENSITIZATION IN RADIOTHERAPY

S. DISCHE, J. F. FOWLER, M. I. SAUNDERS, M. R. L. STRATFORD,
P. ANDERSON, A. I. MINCHINTON AND M. E. LEE

*Marie Curie Research Wing, Gray Laboratory, Mount Vernon Hospital, Northwood, Middlesex HA6 2RN*

Received 27 February 1980 Accepted 28 March 1980

THE RESISTANCE of hypoxic tumour cells to radiotherapy may be a common cause of local failure, for there is good evidence that hypoxic cells exist in nearly all human tumours which are treated. Among the methods which have been introduced to overcome this problem, the chemical radiosensitizers are the most recently developed (Adams *et al.*, 1976). There is considerable interest in them for they have great potential for wide use in radiotherapy (*Lancet*, 1978).

The first drug to be tested clinically for this purpose was metronidazole in 1973, shortly followed in 1974 by misonidazole (Ro 07-582, MISO) a much more effective drug in laboratory studies (Fowler *et al.*, 1976). Now many clinical trials are under way with this compound in Europe, the United States, Canada, South Africa, Australia, New Zealand and Japan.

In the early stages of testing MISO, as the dose was increased, the drug proved to be neurotoxic, producing encephalopathy and peripheral neuropathy (Dische *et al.*, 1977). A dose limit of 12 g/m$^2$ to be given over a period of not less than 17 days has now been generally accepted. With this dose limitation, most cases of peripheral neuropathy are mild and transient, but there is still an incidence of $\sim 30\%$, and an occasional case of more severe toxicity (Dische *et al.*, 1979). The dose may achieve a sensitization of hypoxic cells by a factor of 1·3–1·6 (Fowler *et al.*, 1976) but this must be compared with a factor of 2·5–3 which is required to bring sensitization back to the level of that of oxic cells. Nevertheless there is an expectation that some of the current trials will show benefit with the use of the drug.

A considerable effort is currently being made to develop new compounds which may lead to greater radiosensitization. A direct relationship has been demonstrated between the lipophilicity of radiosensitizing nitroimidazoles and neurotoxicity (Conroy, 1980). High tumour/brain concentration ratios have been obtained with drugs of relatively low lipophilicity (Brown & Lee, 1980). The alternative approach is to develop a compound with a shorter half-life in the plasma, for it has been shown in clinical as well as in animal studies that the area under the time curve of plasma concentration can be directly related to the incidence of neurotoxicity (Dische *et al.*, 1979). The i.v. route has been considered as a potentially superior one, particularly with the use of some of the less lipophilic compounds (White *et al.*, 1980); but this will reduce clinical use because of the practical problems involved with the administration of i.v. preparations, particularly when a patient may receive 20–30 radiation treatments in a course lasting 4–6 weeks.

Desmethylmisonidazole (the Roche experimental drug Ro 05-9963, DESMISO) is the first metabolite of MISO, and it has been detected in the plasma and urine after MISO has been administered (Flockhart *et al.*, 1978*a*). As a radiosensitizer it is about equal in efficiency to MISO (Fowler *et al.*, 1976). The drug, however, combines a lower lipophilicity (the octanol/water partition coefficients are MISO 0·43, DESMISO 0·11) with a shorter half-life in dogs and mice (White & Workman, 1980; Stratford, 1980, personal communication).

Some studies with this compound have, however, suggested similar toxicity to MISO (Roche Products Ltd, 1979). Other studies in mice and dogs have shown less toxicity for DESMISO (Conroy, 1980; Sheldon, 1979, personal communication). Further, recent work with rats at the Institute of Cancer Research using tests of both co-ordination and enzyme analysis of peripheral nerves has shown much lower toxicity with DESMISO than with MISO (Adams, 1980 personal communication).

A prediction of poor absorption of DESMISO when given orally has led to plans for i.v. administration (White & Workman, 1980; Brown & Lee, 1980). A direct testing of DESMISO in man seemed to us the only way to determine the plasma concentration and its time course after administration. Through the kind cooperation of Dr Hassall, Dr Pearson and Dr Lenox-Smith of Roche Products Ltd, a supply of DESMISO was made available to us for a small study of normal volunteers.

The drug was dissolved in water immediately before administration to 3 male subjects. On 15 January 1980, 0·5 g of DESMISO/m² was given under near-fasting conditions to all 3. One week later 1 g of DESMISO/m² was administered to 2 subjects and finally, a week later, a dose of 0·5 g/m² of MISO was administered to all 3. Nitroimidazole concentrations in blood and urine were determined by high-performance liquid chromatography (Dische *et al.*, 1979).

The plasma concentrations measured in the 3 subjects are shown (Figure). The concentrations recorded after administration of MISO are of total nitroimidazoles. The small concentration of DESMISO is thus added to the MISO measured, as they are equally effective radiosensitizers.

FIG. — Desmethylmisonidazole (DESMISO) was administered to 3 male subjects (PA, SD, JFF). The total plasma concentrations of nitroimadazole are shown (μg/ml) for each subject after doses of:

0·5 g DESMISO/m² (▼)

1·0 g DESMISO/m² (▲)

0·5 g MISO/m² (□)

| Drug half lives (L): | ▼ | ▲ | □ |
|---|---|---|---|
| PA | 5·3 | | 9·0 |
| SD | 6·4 | 6·3 | 11·7 |
| JFF | 5·4 | 6·4 | 10·4 |

When we compare the plasma concentrations in the 3 subjects given identical amounts of DESMISO and MISO we find that after 1–2 h the average DESMISO concentrations were 85, 82 and 72%

of the total nitroimidazoles when MISO was given. Subsequently the DESMISO levels fell more rapidly, with half lives of 5·4, 6·4 and 5·3 h compared with 10·4, 11·7 and 9 h for MISO (calculated by least-squares fit). Doubling the dose of DESMISO appears to double the plasma concentrations, as previously observed with MISO (Dische *et al.*, 1979).

Examination of the urine collected in the 24h period after the first administration of DESMISO showed that 44, 55 and 59% of the dose was excreted. After the second administration, when the dose was doubled, 50 and 52% was excreted. In contrast, during the same period after administration of MISO, 31, 23 and 34% was excreted, either unchanged or as DESMISO.

The relationship of plasma concentration to time with DESMISO should lead to satisfactory tumour concentrations (Flockhart *et al.*, 1978*b*). Because of the more rapid clearance, normal tissue exposure will be reduced to half that with MISO. In addition, we can expect a reduction of the concentration in nervous tissue, due to lower lipophilicity.

The results suggest that DESMISO might prove a more efficient drug for oral use as an hypoxic cell sensitizer in man than MISO. Drug concentrations several times greater than those achieved with MISO may be attained and the incidence of neurotoxicity may be reduced. A clinical study to determine drug levels in human tumours and in cerebrospinal fluid is now indicated.

We wish to thank the Medical Research Council and the Cancer Research Campaign for their support of this work and Roche Products Ltd for the supply of desmethylmisonidazole compound.

### REFERENCES

ADAMS, G. E., FOWLER, J. F., DISCHE, S. & THOMLINSON, R. H. (1976) Increased radiation response by chemical sensitization. *Lancet*, i, 186.

BROWN, J. M. & LEE, W. W. (1980) Pharmacokinetic consideration in radiosensitizer development. *Cancer Clin. Trials* (in press).

CONROY, P. J. (1980) Neurotoxicity of hypoxic cell sensitizers. *Cancer Clin. Trials* (in press).

DISCHE, S., SAUNDERS, M. I., LEE, M. E., ADAMS, G. E. & FLOCKHART, I. R. (1977) Clinical testing of the radiosensitizer Ro 07-0582: Experience with multiple doses. *Br. J. Cancer*, **35**, 567.

DISCHE, S., SAUNDERS, M. I., FLOCKHART, I. R., LEE, M. E. & ANDERSON, P. (1979) Misonidazole. A drug for trial in radiotherapy and oncology. *Int. J. Radiat. Oncol. Biol. Phys.*, **5**, 851.

FLOCKHART, I. R., LARGE, P., TROUP, D., MALCOLM, S. L. & MARTEN, T. R. (1978*a*) Pharmacokinetic and metabolic studies of the hypoxic cell radiosensitizer misonidazole. *Xenobiotica*, **8**, 97.

FLOCKHART, I. R., SHELDON, P. W., STRATFORD, I. J. & WATTS, M. E. (1978*b*) A metabolite of the 2-nitroimidazole misonidazole with radiosensitizing properties. *Int. J. Radiat.*, **34**, 91.

FOWLER, J. F., ADAMS, G. E. & DENEKAMP, J. (1976) Radiosensitizers of hypoxic cells in solid tumours. *Cancer Treat. Rev.*, **3**, 227.

LANCET (1978) Hypoxic cell sensitizers. *Lancet*, ii, 617.

WHITE, R. A., BROWN, J. M. & WORKMAN, W. (1980) The pharmacokinetics in the dog of misonidazole and other potentially superior radiosensitizing drugs. *Cancer Clin. Trials* (in press).

WHITE, R. A. S. & WORKMAN, P. (1980) Pharmacokinetic and tumour-penetration properties of the hypoxic cell radiosensitizer desmethylmisonidazole (Ro 05-0063) in dogs. *Br. J. Cancer*, **41**, 268.